# STEPS TOWARD RAISE: CO-DESIGN OF COMPETENCY-BASED EDUCATION TO ENGAGE FAMILY CAREGIVERS AS PARTNERS

**DOI:** 10.1093/geroni/igac059.210

**Published:** 2022-12-20

**Authors:** Jasneet Parmar, Sharon Anderson, Wendy Duggleby, Michelle Lobchuk, Elisabeth Drance, Tanya L'Heureux, Johnna Lowther, Kim Crowder

**Affiliations:** University of Alberta, Edmonton, Alberta, Canada; Faculty of Medicine & Dentistry University of Alberta, Edmonton, Alberta, Canada; University of Alberta, Edmonton, Alberta, Canada; University of Manitoba, Winnipeg, Manitoba, Canada; University of British Columbia, Vancouver, British Columbia, Canada; Department of Family Medicine, University of Alberta, Edmonton, Alberta, Canada; Caregivers Alberta, Edmonton, Alberta, Canada; University of Alberta, Edmonton, Alberta, Canada

## Abstract

Meeting the needs of a growing population of older people living with complex conditions is highly dependent on healthcare providers partnering with family caregivers (FCGs). FCGs provide 90% of the care yet are marginalized within healthcare systems. Educating healthcare providers to support FCGs is a necessary step towards addressing the inconsistent system of supports for diverse FCGs throughout variable care trajectories. Current research suggests that co-design benefits stakeholders, produces superior outcomes, and facilitates moving knowledge efficiently into healthcare practices. Currently, moving best practices into healthcare is a time-consuming process (10-17 years). This presentation will discuss a feasible three-phase co-design process that included 120 multilevel interdisciplinary stakeholders including FCGs, educators, researchers, not-for-profit and healthcare providers/leaders, educational designers, and policy influencers/makers: 1) Developing relationships and insights; 2) Translating insights into education design; and 3) Planning the implementation, spread, and scale-up. The research tools used included literature reviews, qualitative and survey research on specific topics, consultations (symposia, modified Delphi process, co-design meetings), and mixed methods evaluation. Three modules, Foundational, COVID-19, and Advanced have been developed. Learners report high satisfaction, relevance, and significant knowledge gains upon completion. This successful education co-design required three critical elements: 1) an engaged co-design team led by people knowledgeable about healthcare and FCGs; 2) team access to collaborators/staff with the appropriate theoretical, research, and facilitation skills; and 3) an educational design team to bring stakeholders’ ideas to life. Leveraging stakeholders’ insights are a critical step towards the RAISE act goal of educating healthcare providers to include FCGs as partners-in-care.

